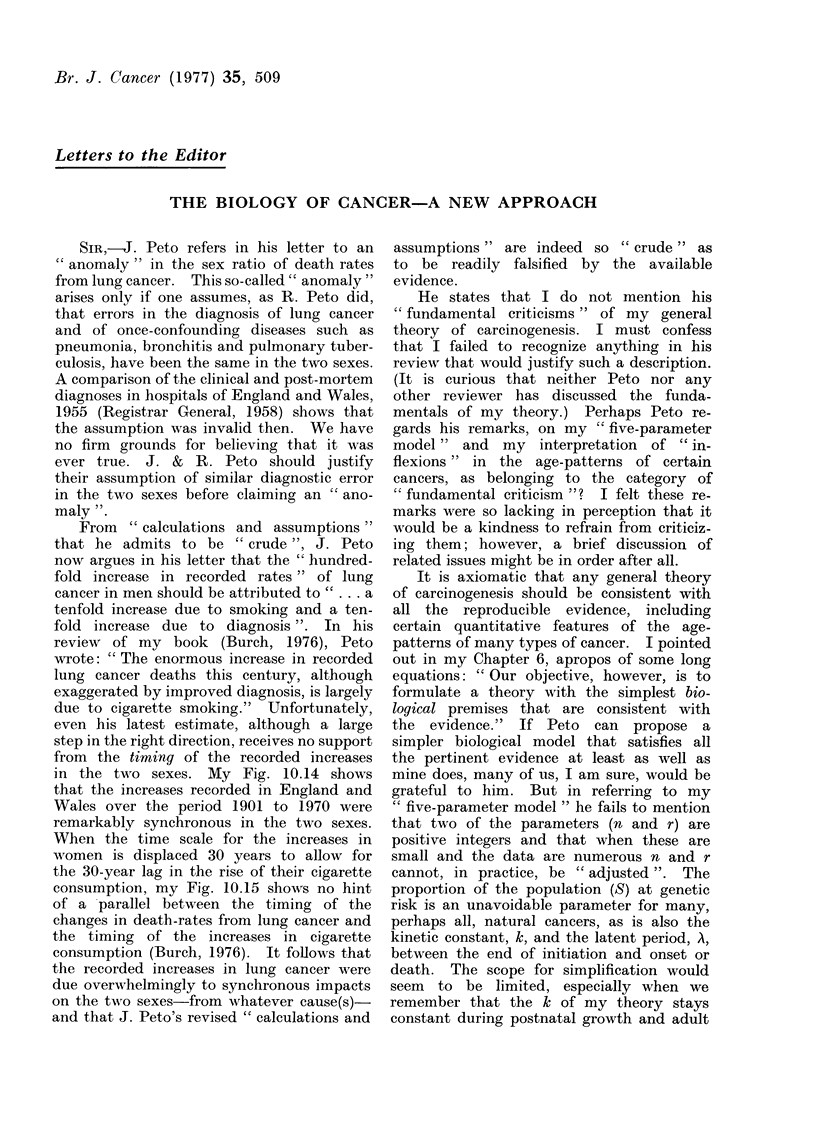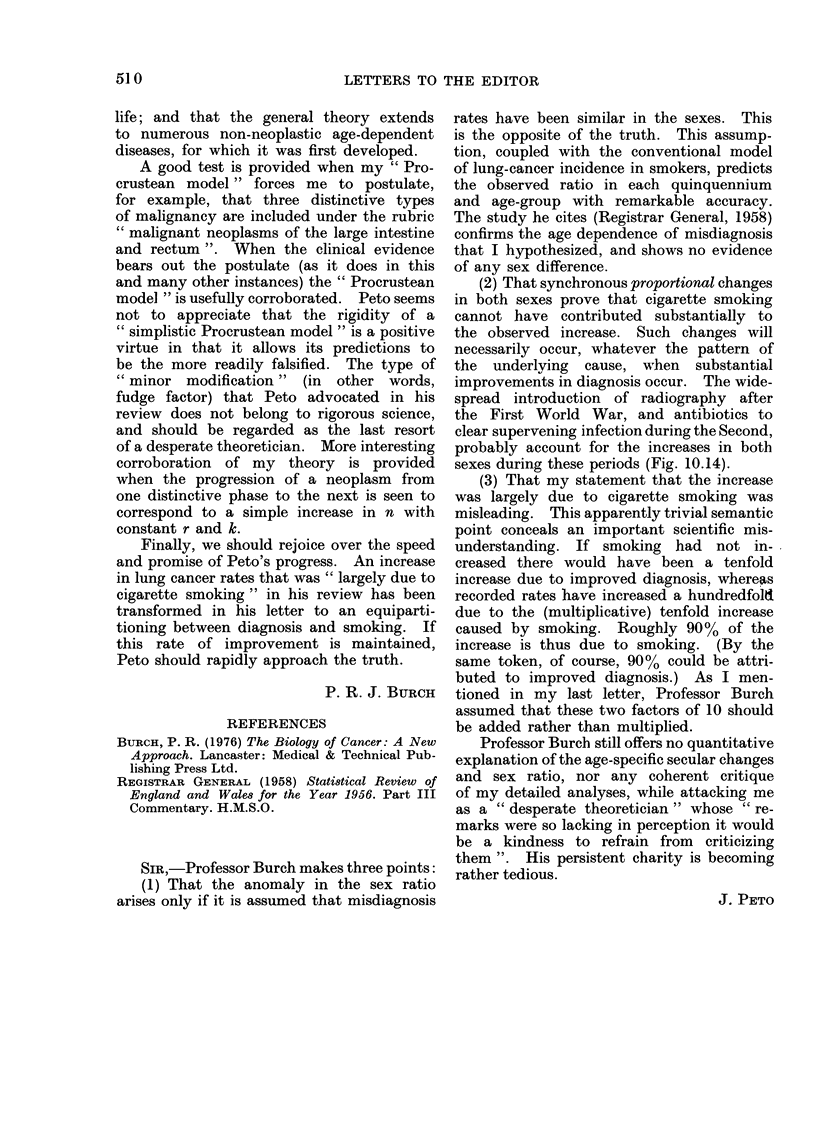# The Biology of Cancer—A New Approach

**Published:** 1977-04

**Authors:** P. R. J. Burch


*Br. J. Cancer* (1977) **35**, 509

### *Letters to the Editor*

### THE BIOLOGY OF CANCER—A NEW APPROACH

SIR,—J. Peto refers in his letter to an " anomaly " in the sex ratio of death rates from lung cancer. This so-called " anomaly " arises only if one assumes, as R. Peto did, that errors in the diagnosis of lung cancer and of once-confounding diseases such as pneumonia, bronchitis and pulmonary tuberculosis, have been the same in the two sexes. A comparison of the clinical and post-mortem diagnoses in hospitals of England and Wales, 1955 (Registrar General, 1958) shows that the assumption was invalid then. We have no firm grounds for believing that it was ever true. J. & R. Peto should justify their assumption of similar diagnostic error in the two sexes before claiming an " anomaly ".

From " calculations and assumptions " that he admits to be " crude ", J. Peto now argues in his letter that the " hundred-fold increase in recorded rates " of lung cancer in men should be attributed to " . . . a tenfold increase due to smoking and a tenfold increase due to diagnosis ". In his review of my book (Burch, 1976), Peto wrote: " The enormous increase in recorded lung cancer deaths this century, although exaggerated by improved diagnosis, is largely due to cigarette smoking." Unfortunately, even his latest estimate, although a large step in the right direction, receives no support from the *timing* of the recorded increases in the two sexes. My Fig. 10.14 shows that the increases recorded in England and Wales over the period 1901 to 1970 were remarkably synchronous in the two sexes. When the time scale for the increases in women is displaced 30 years to allow for the 30-year lag in the rise of their cigarette consumption, my Fig. 10.15 shows no hint of a parallel between the timing of the changes in death-rates from lung cancer and the timing of the increases in cigarette consumption (Burch, 1976). It follows that the recorded increases in lung cancer were due overwhelmingly to synchronous impacts on the two sexes—from whatever cause(s)—and that J. Peto's revised " calculations and assumptions " are indeed so " crude " as to be readily falsified by the available evidence.

He states that I do not mention his " fundamental criticisms " of my general theory of carcinogenesis. I must confess that I failed to recognize anything in his review that would justify such a description. (It is curious that neither Peto nor any other reviewer has discussed the fundamentals of my theory.) Perhaps Peto regards his remarks, on my " five-parameter model " and my interpretation of " inflexions " in the age-patterns of certain cancers, as belonging to the category of " fundamental criticism "? I felt these remarks were so lacking in perception that it would be a kindness to refrain from criticizing them; however, a brief discussion of related issues might be in order after all.

It is axiomatic that any general theory of carcinogenesis should be consistent with all the reproducible evidence, including certain quantitative features of the age-patterns of many types of cancer. I pointed out in my Chapter 6, apropos of some long equations: " Our objective, however, is to formulate a theory with the simplest *biological* premises that are consistent with the evidence." If Peto can propose a simpler biological model that satisfies all the pertinent evidence at least as well as mine does, many of us, I am sure, would be grateful to him. But in referring to my " five-parameter model " he fails to mention that two of the parameters ($n$ and $r$) are positive integers and that when these are small and the data are numerous $n$ and $r$ cannot, in practice, be " adjusted ". The proportion of the population ($S$) at genetic risk is an unavoidable parameter for many, perhaps all, natural cancers, as is also the kinetic constant, $k$, and the latent period, $\lambda$, between the end of initiation and onset or death. The scope for simplification would seem to be limited, especially when we remember that the $k$ of my theory stays constant during postnatal growth and adult life; and that the general theory extends to numerous non-neoplastic age-dependent diseases, for which it was first developed.

A good test is provided when my " Procrustean model " forces me to postulate, for example, that three distinctive types of malignancy are included under the rubric " malignant neoplasms of the large intestine and rectum ". When the clinical evidence bears out the postulate (as it does in this and many other instances) the " Procrustean model " is usefully corroborated. Peto seems not to appreciate that the rigidity of a " simplistic Procrustean model " is a positive virtue in that it allows its predictions to be the more readily falsified. The type of " minor modification " (in other words, fudge factor) that Peto advocated in his review does not belong to rigorous science, and should be regarded as the last resort of a desperate theoretician. More interesting corroboration of my theory is provided when the progression of a neoplasm from one distinctive phase to the next is seen to correspond to a simple increase in $n$ with constant $r$ and $k$.

Finally, we should rejoice over the speed and promise of Peto's progress. An increase in lung cancer rates that was " largely due to cigarette smoking " in his review has been transformed in his letter to an equipartitioning between diagnosis and smoking. If this rate of improvement is maintained, Peto should rapidly approach the truth.

P. R. J. Burch

REFERENCES

Burch, P. R. (1976) *The Biology of Cancer: A New Approach.* Lancaster: Medical & Technical Publishing Press Ltd.

Registrar General (1958) *Statistical Review of England and Wales for the Year 1956.* Part III Commentary. H.M.S.O.